# Supplementary figures and images for: Genetic Insights into Intestinal Microbiota and Risk of Infertility: A Mendelian Randomization Study
Source: Microorganisms. 2023 Sep 15;11(9):2319. doi: 10.3390/microorganisms11092319 (PMC10538041; doi:10.3390/microorganisms11092319)

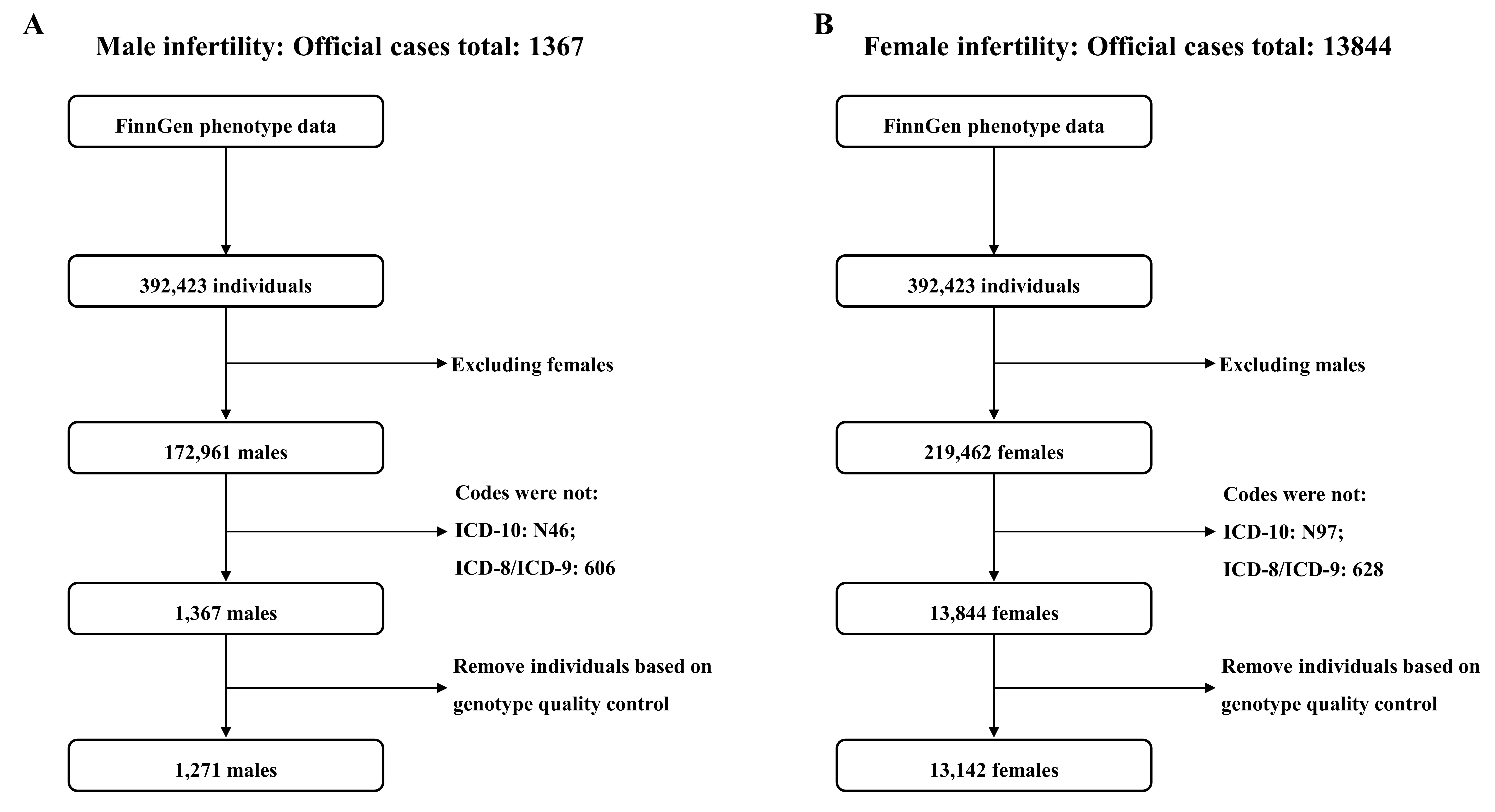

Supplement: Supplementary file 1 [file microorganisms-11-02319-s001.zip › Figure S1 Flow chart.jpg]

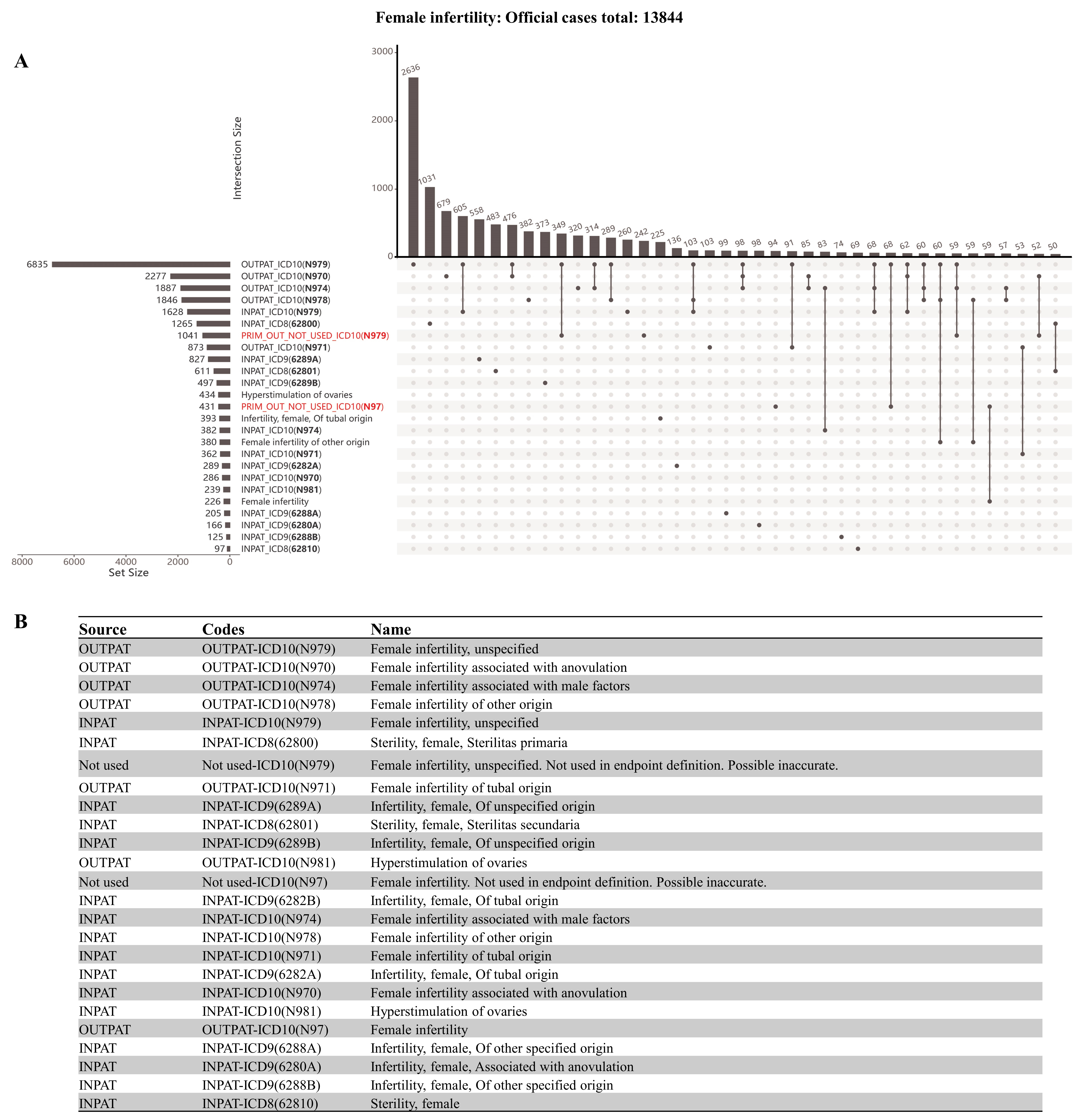

Supplement: Supplementary file 1 [file microorganisms-11-02319-s001.zip › Figure S2 Female.jpg]

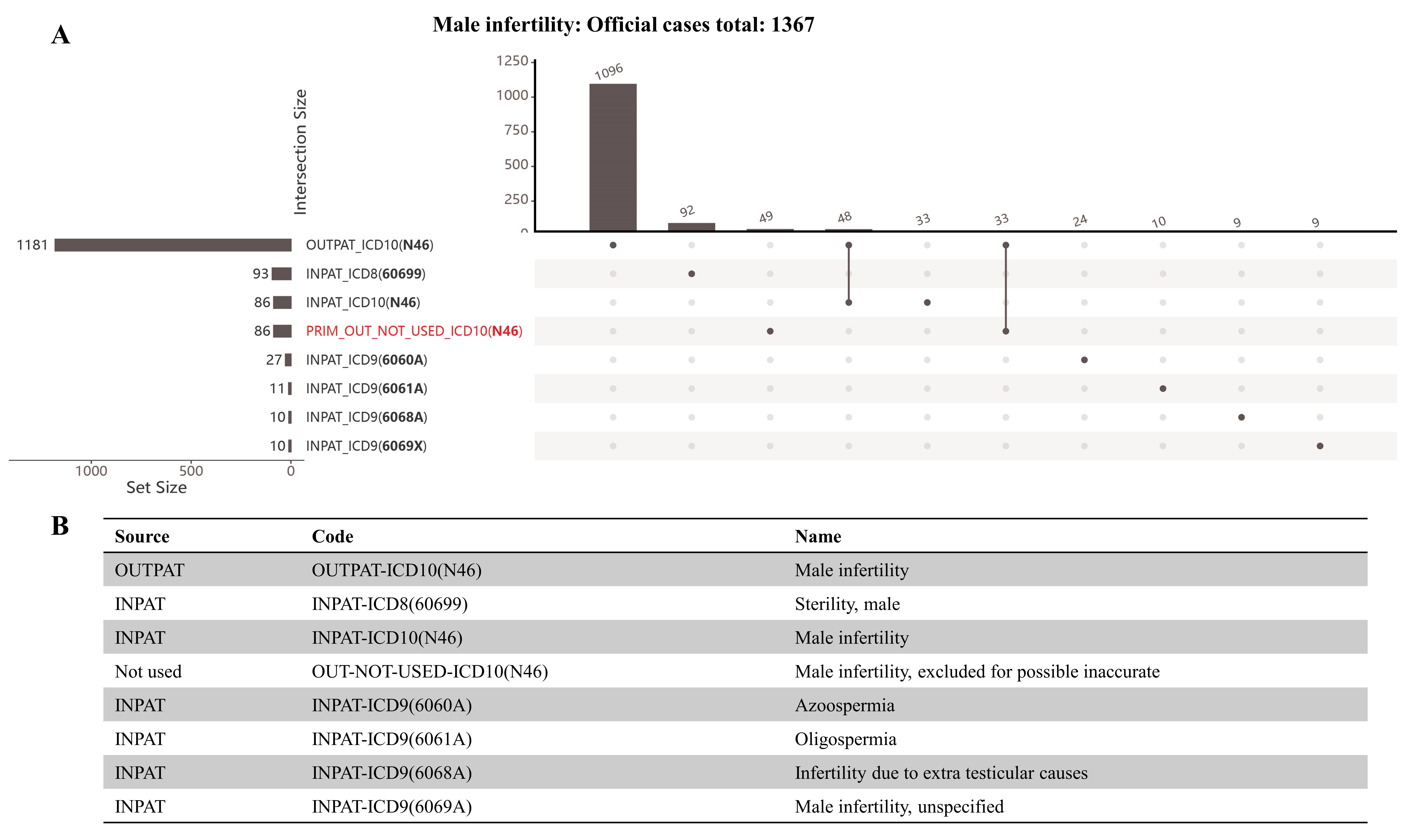

Supplement: Supplementary file 1 [file microorganisms-11-02319-s001.zip › Figure S3 Male.jpg]
